# Precise energy modeling and green retrofitting optimization of existing buildings based on BIM and deep learning approaches

**DOI:** 10.1371/journal.pone.0337469

**Published:** 2025-12-12

**Authors:** Xiyang Ge, Sharifah Akmam Syed Zakaria, Chenyu Wang, Meng Zhu

**Affiliations:** 1 School of Civil Engineering, Universiti Sains Malaysia, Penang, Malaysia; 2 School of Architectural Engineering, Jiaxing Nanhu University, Jiaxing, China; University of Tehran, IRAN, ISLAMIC REPUBLIC OF

## Abstract

The construction industry has emerged as a major contributor to global energy consumption and greenhouse gas emissions amidst continuously rising worldwide energy demands. Enhancing building energy efficiency represents a critical intervention for achieving energy conservation and emission reduction targets. In the context of smart city development, such optimization efforts provide substantial momentum for sustainable urban growth. This study introduces a novel methodology that integrates Transformer models with Graph Neural Networks (GNNs) to improve the accuracy and operability of building energy efficiency prediction through advanced deep learning techniques. By leveraging Building Information Modeling (BIM) data to model spatial structures and energy consumption patterns, GNNs effectively capture complex relationships between building components, thereby strengthening the characterization of multidimensional interactions within structures. The self-attention mechanism in Transformers enables the model to focus on key factors such as energy consumption hotspots and temporal variations, enhancing learning capabilities across both spatial and temporal dimensions. To further augment optimization performance, we incorporate Generative Adversarial Networks (GANs) to generate diverse green renovation schemes, expanding optimization pathways and enhancing model adaptability and robustness. Experimental validation using BIM data demonstrates that our integrated approach outperforms traditional energy efficiency optimization models, increasing energy savings by nearly 4 These findings establish that BIM data-integrated deep learning optimization methodologies offer significant potential for providing effective energy efficiency prediction and optimization decision support. Such approaches directly contribute to building design and operations in smart cities, advancing the realization of green buildings and sustainable urban development.

## 1 Introduction

With the ongoing global warming, climate change is getting more severe [1]. The construction industry, a major energy consumer and emitter of greenhouse gases, faces great pressure in energy - saving and emissions - reduction [2]. Under the smart city development background, improving building energy efficiency and cutting carbon emissions is key to sustainable development and vital for promoting the green urban transformation [3]. Optimizing building energy efficiency can lower energy consumption and greenhouse gas emissions, ease environmental burdens, enhance residents’ quality of life, and drive sustainable economic growth. In smart cities, building energy - efficiency optimization goes far beyond simple energy - saving; it’s closely tied to overall urban resource management, transport planning, air - quality control, etc. Traditionally, building energy - efficiency optimization depended on empirical or rule - based models [4]. Yet, when dealing with complex building data and multi - dimensional optimization problems in dynamic environments, these conventional methods show clear limitations. Their prediction accuracy is poor, and they can’t fully account for the complex interactions between buildings and the urban environment. Thus, in smart cities, more advanced methods are needed for building energy - efficiency optimization to tackle these challenges.

Deep learning [5] and artificial intelligence [6] technologies are advancing rapidly, significantly accelerating building energy efficiency optimization. Within the context of smart city development, deep learning demonstrates distinct advantages through its ability to automatically extract features from large-scale datasets and accurately identify latent energy consumption patterns, thereby offering more precise and adaptable solutions. The integration of deep learning with BIM [7] and sensor data enables the development of high-precision modeling approaches for building energy efficiency enhancement. This integration not only substantially improves the accuracy of building energy efficiency predictions but also supports real-time energy management systems, facilitating data-driven decision-making processes while contributing to the intelligent management and optimization of comprehensive urban energy systems. In contemporary building energy efficiency optimization research, particularly within the smart city framework, methodological approaches can be classified into four distinct paradigms. Each paradigm exhibits unique characteristics with corresponding advantages and limitations. These approaches face numerous critical challenges within the complex and heterogeneous demand scenarios of smart cities, while simultaneously presenting unprecedented opportunities for advancement.

- Traditional Modeling Methods: Traditional building energy efficiency optimization methods primarily rely on physics-based energy simulations and empirical regression models [8]. Energy simulation tools such as EnergyPlus [9] and TRNSYS [10] predict building energy efficiency by simulating heat transfer, airflow, and other environmental factors. However, these approaches often require substantial computational resources and demonstrate limitations when handling complex building data and nonlinear relationships. Empirical regression models establish linear correlations between energy efficiency and building parameters (e.g., area, window-to-wall ratio) based on physical characteristics. While computationally efficient to implement, these models typically exhibit limited accuracy and inadequate performance when processing multidimensional data.
- Machine Learning Methods: The advancement of machine learning techniques has introduced algorithms such as Support Vector Machines (SVM) [11], Random Forests (RF) [12], and K-Nearest Neighbors (KNN) [13] to building energy efficiency optimization. These algorithms learn from data patterns to predict building energy performance. SVM constructs hyperplanes in high-dimensional feature space, making it suitable for energy efficiency classification and regression tasks. Random Forest enhances prediction stability and accuracy by integrating multiple decision trees. KNN generates predictions based on similar samples, proving effective for energy efficiency prediction with smaller datasets. Additionally, multi-objective energy efficiency optimization problems benefit from algorithms such as genetic algorithms and particle swarm optimization, which efficiently explore optimal solutions in building design parameters.
- Deep Learning Methods: Deep learning approaches demonstrate unique advantages when processing large-scale, high-dimensional building data. Convolutional Neural Networks (CNN) [14] effectively analyze building design drawings and spatial layouts, extracting features that enhance energy efficiency prediction accuracy through improved spatial modeling. Recurrent Neural Networks [15] and Long Short-Term Memory networks [16] excel at processing time-varying building energy data, making them particularly suitable for real-time prediction applications. Autoencoders extract key features from high-dimensional data, thereby simplifying energy efficiency model construction. Generative Adversarial Networks [17] offer significant advantages in generating diverse green retrofitting plans, substantially increasing model adaptability through the production of varied energy efficiency optimization schemes.
- Hybrid Methods: Hybrid approaches integrate the strengths of conventional modeling techniques with modern machine learning and deep learning methods to enhance both accuracy and efficiency in building energy optimization. The combination of Building Information Modeling (BIM) with deep learning enables more precise representation of building spatial structures and energy consumption patterns. Multi-objective optimization methods incorporate genetic algorithms, particle swarm optimization, and deep learning techniques [18] to balance multiple optimization objectives simultaneously, thereby improving overall energy efficiency outcomes while addressing the complex requirements of contemporary building systems.

Despite the progress made in existing research, the field of building energy efficiency optimization still faces numerous challenges [19]. First, the complexity and diversity of building data make it difficult for existing models to comprehensively capture the various interactions within buildings. Second, current methods often focus on optimizing only specific aspects of building energy efficiency (such as building façades, HVAC systems, etc.), lacking a holistic, systematic optimization approach. Additionally, traditional methods and machine learning techniques still face limitations when dealing with large-scale, dynamically changing data, especially in considering the variable energy consumption patterns during the building’s usage.

This study addresses critical limitations in smart city development, specifically in building energy efficiency optimization. We propose an innovative approach that integrates deep learning techniques with BIM data to enhance building energy performance. As smart cities evolve, optimizing building energy efficiency impacts not only individual building consumption patterns but also encompasses broader urban resource management and environmental sustainability. Our method significantly improves the accuracy of building energy efficiency prediction, thereby providing real-time intelligent decision support throughout the design, operation, and maintenance phases. This advancement promotes green building development while contributing to global energy conservation and carbon reduction objectives. Specifically, we combine BIM data with GNNs to construct comprehensive models of building spatial layouts and capture complex interdependencies among diverse building components. Furthermore, by incorporating the self-attention mechanism from Transformer architectures, we enhance the model’s learning capabilities across both temporal and spatial dimensions. This approach enables precise identification of energy consumption hotspots and temporal variations, facilitating targeted energy efficiency optimization. The proposed methodology not only reduces energy waste and carbon emissions but also offers innovative building energy management solutions for smart cities, thereby accelerating green transformation and sustainable urban development.

Building energy efficiency optimization is essential for smart city development and plays a critical role in achieving global emission reduction and sustainable development goals. Despite progress in existing optimization approaches, traditional methods face increasing challenges due to the expanding scale and complexity of smart cities. This study proposes a novel building energy efficiency optimization framework that integrates self-attention mechanisms, GNNs, and GANs. By leveraging these deep learning architectures to extract comprehensive spatial and temporal information from BIM data, our approach significantly enhances prediction accuracy and generates diverse optimization solutions for smart urban environments. Implementation of this framework demonstrably improves building energy efficiency management throughout both design and operational phases of smart cities, thereby providing robust support for green development initiatives and sustainable urban futures. The integrated approach not only addresses current limitations in building energy modeling but also establishes a foundation for future intelligent building management systems that can adapt to evolving urban complexities.

During the study, we found our main contributions in three aspects:

1. Our first contribution is the application of GNNs to building energy efficiency optimization. Traditional methods predominantly rely on static linear models that fail to capture the complex spatial and functional relationships within building systems. GNNs, through graph-based modeling of nodes and edges, effectively learn the intricate interactions among building components. This approach accurately identifies key energy-efficiency factors, providing superior guidance for optimization strategies. This methodology expands research perspectives and addresses energy efficiency challenges in complex architectural scenarios.

2. The second contribution is the integration of the self-attention mechanism from Transformer models to enhance the accuracy of building energy efficiency prediction. Conventional approaches demonstrate limitations in capturing temporal patterns of energy consumption in time-series data. The self-attention mechanism dynamically focuses on and weights key factors across temporal dimensions, significantly improving prediction accuracy. Additionally, the model effectively processes long-sequence data, increasing its applicability in actual building operations and providing robust support for real-time energy efficiency optimization. This innovation enriches building energy efficiency prediction methodology, offering distinct advantages in complex time-dependent scenarios.

3. The third innovation is the implementation of Generative Adversarial Networks (GANs) to generate eco-renovation plans for building energy efficiency optimization. By producing diverse, high-quality renovation strategies, GANs expand optimization pathways and enhance model adaptability and robustness. Unlike traditional rule-based methods, GANs generate customized plans derived from real-world data patterns. This approach facilitates optimal building energy efficiency across diverse environmental conditions and provides more flexible management solutions.

The research is structured as follows: Sect 2, the “Literature Review,” offers a comprehensive retrospective of the current research in building energy efficiency optimization, delves into the application status of BIM technology and deep learning algorithms, highlights the deficiencies in existing studies, and clarifies the innovations and significance of this research. Sect 3, “Research Methods,” thoroughly explains the three deep learning architectures central to this study—GNN, Transformer, and GANs— and explores their collaborative mechanisms to enhance building energy efficiency accurately and efficiently. Sect 4, the “Experimental Section,” comprehensively presents the experimental design and datasets, covering the experimental setup, the rationale for selecting evaluation metrics, and the preprocessing of datasets. Through numerous comparative and ablation experiments, it demonstrates the effectiveness of the proposed method. Lastly, Sect 5, “Conclusion and Outlook,” summarizes the key findings and contributions of the research, examines its limitations, and suggests future research directions, providing new insights and methodological support for improving building energy efficiency and advancing green building development.

## 2 Related work

### 2.1 Historical evolution of energy efficiency optimization

The pursuit of building energy efficiency has progressed through several distinct technological stages. Early efforts (1980s–2000s) relied on physics-based simulation engines such as DOE-2 [20] and TRNSYS [21]. These tools required extensive manual parameterization and offered limited interoperability with other information systems. From roughly 2000 to 2010, pioneering attempts were made to link Building Information Modeling (BIM) with building performance simulation. Augenbroe [22] laid conceptual and methodological foundations for coupling building information systems with performance analysis, while Bazjanac [23] advanced Industry Foundation Classes (IFC)-based data exchange, streamlining the transfer from BIM to Building Energy Modeling. Over the past decade (2010–present), the field has increasingly embraced data-driven methodologies, embedding machine learning and, more recently, deep learning within BIM-centric workflows. Scientometric analyses reflect a pronounced rise in BIM-for-energy research after 2016, with a shift from primarily theoretical studies toward practical applications and the integration of emerging technologies such as the Internet of Things (IoT) and artificial intelligence (AI).

### 2.2 BIM-driven energy optimization approaches

To address building energy efficiency across the full life cycle—design, construction, and operation—researchers have increasingly adopted BIM as a digital backbone for analysis and decision-making. In the context of smart cities and the accelerating Fourth Industrial Revolution [24], BIM is converging with IoT [25], Big Data [26], and AI, opening new avenues for reducing energy consumption, improving operational efficiency, and mitigating environmental impacts. These convergent technologies enhance the energy performance of individual buildings and, through city-scale data integration and intelligent management, support broader goals for green and sustainable urban development [27–29].

Several reviews have mapped this evolving landscape and identified future directions. Begić et al. [30] synthesized the Building 4.0 concept and analyzed the potential of integrating BIM with IoT and Big Data, concluding that such fusion can greatly accelerate digitalization, especially in early project stages. Pereira et al. [31] surveyed BIM’s role in enhancing energy performance across the building life cycle, highlighting its effectiveness for simulation-driven diagnosis and optimization in the design and construction phases [32].

Beyond reviews, empirical and methodological contributions further illustrate BIM’s impact. Xu, Fang et al. [33] proposed a BIM–convolutional neural network hybrid model for energy-efficiency optimization; experiments showed meaningful reductions in energy use along with improvements in daylighting and ventilation, underscoring the promise of coupling deep learning with BIM. Durdyev, Serdar et al. [34] examined BIM-enabled energy evaluation and found that integrating BIM with life-cycle assessment (LCA) improves the rigor of energy and carbon accounting. Kusi, Elijah et al. [35] used BIM to compare traditional and green buildings, demonstrating substantial reductions in energy consumption and emissions for green designs and thereby emphasizing BIM’s role in sustainable design. Complementing these studies, Maedeh et al. [36] presented a framework uniting mathematical optimization, 6D BIM, and LCA to identify cost- and performance-optimal retrofit strategies, illustrating BIM’s strength in supporting retrofit decision-making [37].

### 2.3 Non-BIM methodologies and theoretical foundations

A comprehensive understanding of building energy efficiency also draws on methodologies that operate outside traditional BIM-centric pipelines. Lu [38] developed a cost-efficient IoT-driven monitoring system that delivers real-time energy diagnostics without BIM infrastructure, achieving accuracy comparable to BIM-based approaches. Zhang et al. [39] introduced surrogate modeling strategies that substantially reduce the computational burden of energy simulations relative to conventional BIM-based workflows, enabling faster design iteration and broader parametric exploration [40,41].

### 2.4 Critical gaps and research opportunities

Despite notable advances, current research often bifurcates into BIM-driven versus non-BIM approaches, with limited work on cohesive frameworks that exploit the strengths of both. Most BIM-based optimization remains design-centric and does not readily adapt to operational-phase dynamics. Without robust mechanisms for continuous data assimilation, BIM models are prone to model-to-reality drift (sometimes termed digital decay), diminishing their long-term value for optimization. Moreover, while theories from complex systems and socio-technical systems offer explanatory power for multi-actor, multi-scale processes, their operationalization within BIM-enabled toolchains is still nascent. This gap is especially evident in the limited treatment of human–building interaction dynamics, which are critical for realizing and sustaining energy performance in actual use.

## 3 Methodology

**Fig 1:**
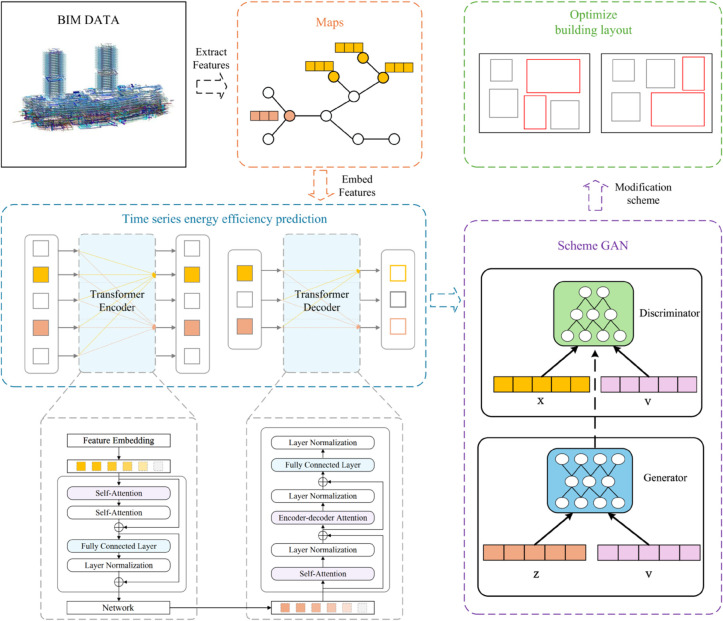
Flowchart of building energy efficiency optimization based on BIM data and deep learning.

This section presents our integrated framework for building energy efficiency optimization that synergistically combines Graph Neural Networks, Transformer models, and Generative Adversarial Networks. Our method establishes a unified pipeline where GNNs capture complex spatial dependencies among building components through heterogeneous graph representations, Transformers model temporal energy consumption patterns from time series data, and GANs generate diverse optimization solutions based on the predicted energy profiles. The synergistic effect of these three modules at different stages provides an efficient and comprehensive framework for energy efficiency optimization. Fig 1 illustrates the overall workflow of our proposed approach.

### 3.1 BIM data preprocessing and graph construction

We construct heterogeneous building graphs from BIM models to capture both structural and functional relationships within buildings. In our graph representation, nodes represent two types of entities: physical spaces such as rooms, corridors, and zones, and mechanical systems including HVAC equipment, lighting fixtures, and control devices. Edges encode three categories of relationships: spatial adjacency between rooms sharing walls, floors, or ceilings; system to space connections indicating which equipment serves which zones; and system to system interactions representing ductwork, piping networks, or control dependencies.

Node features are extracted from BIM data and include geometric properties (volume, surface area, orientation, window to wall ratio), thermal characteristics (U values for walls and windows, thermal mass, insulation levels), and operational parameters (temperature setpoints, occupancy schedules, equipment capacities). Edge features capture connection types, flow capacities, thermal resistance, and control relationships. This graph representation preserves the building’s structural and functional organization while remaining amenable to neural network processing through sparse adjacency matrices and mini batch sampling.

### 3.2 GNN based spatial modeling architecture

Our spatial model employs a three layer Edge Conditioned Convolution (ECC) architecture specifically designed for heterogeneous building graphs. Each layer uses 128 dimensional node embeddings to balance expressiveness and computational efficiency. We incorporate residual connections between layers to facilitate gradient flow during training, particularly important given the depth of our network. Batch normalization is applied after each convolution operation to stabilize learning dynamics and improve convergence speed. To prevent overfitting on building specific patterns, we apply dropout with probability 0.2 during training.

The ECC operation is particularly suitable for our heterogeneous building graphs because edge features can modulate the aggregation of neighbor information. This capability is critical for distinguishing between different types of building connections, such as direct thermal conduction through shared walls versus indirect coupling through HVAC distribution networks. After the three GNN layers process the local neighborhood information, we apply global attention pooling to generate a fixed size graph level representation. This pooling mechanism learns to weight nodes based on their importance for energy prediction rather than treating all building components equally, allowing the model to focus on critical energy consumers and thermal bridges.

**Fig 2:**
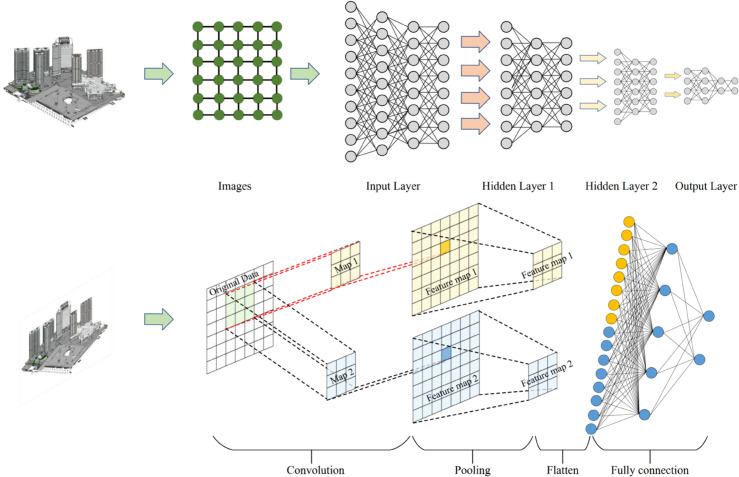
Schematic of GNN architecture in building energy efficiency optimization.

The pooled graph representation feeds into a multi layer perceptron with two hidden layers of dimensions 256 and 128, followed by a final output layer. This MLP head predicts both total building energy consumption and sub meter consumption for major end uses including HVAC systems, lighting, and plug loads. The multi output architecture enables the model to learn more refined representations of energy distribution patterns within buildings. Fig 2 illustrates our GNN architecture for building energy efficiency optimization.

The spatial model is trained to minimize a composite loss function that balances overall prediction accuracy with sub meter granularity. We use Mean Squared Error (MSE) as the primary loss but augment it with an auxiliary MSE loss on sub meter predictions weighted by 0.3. This multi task learning approach improves the model’s ability to understand how energy is distributed across different building systems:

$${ℒ}_{\text{spatial}}=\text{MSE}(E_{\text{total}},{\hat{E}}_{\text{total}})+0.3\sum_{i=1}^{N_{\text{sub}}}\text{MSE}(E_{\text{sub},i},{\hat{E}}_{\text{sub},i})$$ (1)

where $E_{\text{total}}$ and ${\hat{E}}_{\text{total}}$ denote true and predicted total energy consumption, while $E_{\text{sub},i}$ and ${\hat{E}}_{\text{sub},i}$ represent true and predicted consumption for the *i*th submeter category, and $N_{\text{sub}}$ is the number of submeter categories tracked.

### 3.3 Transformer based temporal modeling architecture

To capture temporal dynamics of building energy consumption, we implement a two layer Transformer encoder that processes multivariate time series data. Unlike traditional recurrent architectures, our Transformer based approach enables parallel processing of temporal sequences and effectively models long range dependencies in energy consumption patterns influenced by weather conditions, occupancy schedules, and operational strategies.

The input to our temporal model consists of aligned time series measurements including energy consumption values and environmental variables such as outdoor temperature, humidity, and solar radiation. We support two temporal granularities: hourly sampling with sequence length 168 corresponding to one week of data, and daily sampling with sequence length 60 representing two months of observations. Each timestep is embedded into a 128 dimensional vector through a learned linear projection that maps the raw input features to the model’s internal representation space.

Our Transformer encoder architecture consists of two stacked layers, each containing multi head self attention and position wise feed forward networks. We employ four attention heads per layer, allowing the model to simultaneously attend to different aspects of temporal patterns such as daily cycles, weekly trends, and weather correlations. The attention mechanism computes relationships between all timesteps in the input sequence, enabling the model to identify which historical periods are most relevant for predicting future consumption.

Within each Transformer layer, the feed forward network has an expansion factor of 2, projecting representations from 128 dimensions to 256 dimensions through the first linear layer before contracting back to 128 dimensions in the second layer. We apply ReLU activation between these transformations to introduce nonlinearity. To prevent overfitting on building specific temporal patterns, we incorporate dropout with probability 0.1 within both the attention computations and feed forward layers.

We adopt the pre normalization configuration where layer normalization is applied before each sub block rather than after, which we found provides more stable training dynamics compared to the post normalization variant. Residual connections are added around both the attention and feed forward sub blocks to facilitate gradient propagation through the network depth.

**Fig 3:**
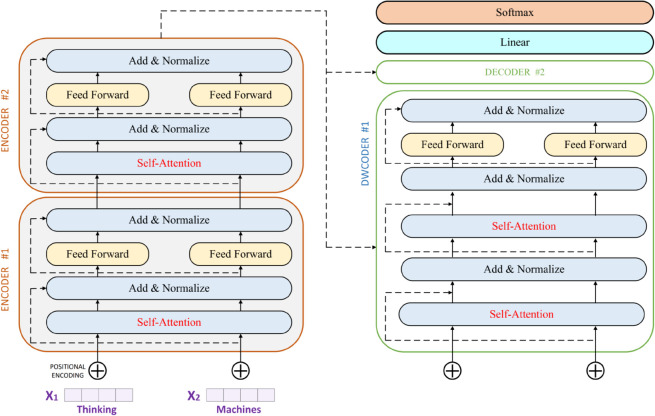
Architecture of the transformer based temporal model.

The Transformer processes the entire input sequence but we only retain the final timestep’s hidden representation for regression, implementing a sequence to one prediction paradigm. This representation captures the temporal context necessary for forecasting and is projected through a single linear layer to predict the next period’s energy consumption. Fig 3 illustrates the architecture of our temporal modeling component.

The temporal model is optimized using Mean Squared Error loss between predicted and actual energy consumption at the next timestep:

$${ℒ}_{\text{temporal}}=\frac{1}{N}\sum_{i=1}^{N}{\left({\hat{E}}_{t+1}^{\left(i\right)}-E_{t+1}^{\left(i\right)}\right)}^{2}$$ (2)

where ${\hat{E}}_{t+1}^{\left(i\right)}$ is the predicted energy consumption for sample *i* at time *t*  +  1, $E_{t+1}^{\left(i\right)}$ is the actual consumption, and *N* is the number of samples in the training batch. We train the temporal model using the AdamW optimizer with learning rate 1 × 10^−3^, weight decay 1 × 10^−4^, and the standard beta parameters (0.9, 0.999) for adaptive moment estimation.

### 3.4 Generative adversarial networks

**Fig 4:**
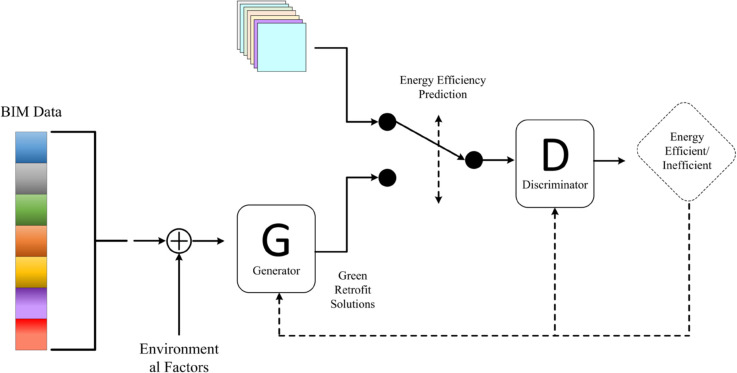
Application framework of Generative Adversarial Network (GAN) in building energy efficiency optimization.

Proposed by Goodfellow et al. in 2014, Generative Adversarial Networks (GANs) [42] are an innovative deep learning architecture involving two neural networks: the generator and the discriminator. The generator learns from training data to create realistic samples, while the discriminator distinguishes between real and generated samples. Through adversarial training, the generator continuously improves based on feedback from the discriminator, aiming to produce samples indistinguishable from real data. This process results in high-quality, authentic-looking samples. Fig 4 illustrates the GAN framework for building energy efficiency optimization.

In building energy efficiency prediction and green renovation scheme generation, GAN can be used to generate building renovation plans that meet the target energy efficiency standards. By combining with the previously mentioned Graph Neural Network and Transformer models, the generated building renovation schemes can be optimized based on the energy efficiency prediction results to achieve energy-saving and green building design goals. This adversarial game is described by the following loss function:

$${ℒ}_{GAN}(D,G)={𝔼}_{x\sim p_{data}(x)}[\log D(x)]+{𝔼}_{z\sim p_{z}(z)}[\log(1-D(G(z)))]$$ (3)

$p_{\text{data}}(x)$ represents the distribution of real data, reflecting the statistical characteristics of the actual dataset. This is the target distribution that the generator aims to learn and mimic during model training. *p*_*z*_(*z*) denotes the input noise distribution for the generator, typically a standard normal distribution. This allows the generator to learn effective data patterns from random noise, producing diverse samples. *D*(*x*) is the discriminator’s predicted probability that sample *x* is from the real data, ranging between 0 and 1. When *D*(*x*) is close to 1, the discriminator deems *x* highly likely to be real. *G*(*z*) refers to the samples generated by the generator from input noise *z*, designed to resemble the real data distribution and deceive the discriminator.

The generator’s objective is to minimize the discriminator’s classification result *D*(*G*(*z*)), such that the generated sample is classified as real data by the discriminator. Therefore, the generator’s loss function is:

$${ℒ}_{G}=-{𝔼}_{z\sim p_{z}(z)}[\log D(G(z))]$$ (4)

The loss function of the discriminator is:

$${ℒ}_{D}=-{𝔼}_{x\sim p_{data}(x)}[\log D(x)]-{𝔼}_{z\sim p_{z}(z)}[\log(1-D(G(z)))]$$ (5)

During the training process, the generator and discriminator are updated alternately. By optimizing the loss functions, the generator aims to produce realistic samples, while the discriminator tries to distinguish between real and fake samples as accurately as possible. The specific optimization process is carried out using the following gradient descent update rule:

1. Update the discriminator: Train the discriminator using both real samples and generated fake samples, updating the discriminator’s parameters $\theta_{D}$:

$$\theta_{D}\leftarrow \theta_{D}-\eta_{D}\nabla_{\theta_{D}}{ℒ}_{D}$$ (6)

2. Update the generator: Train the generator using the generated samples, updating the generator’s parameters $\theta_{G}$:

$$\theta_{G}\leftarrow \theta_{G}-\eta_{G}\nabla_{\theta_{G}}{ℒ}_{G}$$ (7)

Where $\eta_{D}$ and $\eta_{G}$ are the learning rates for the discriminator and the generator, respectively.

Through the adversarial training process between the generator and discriminator, Generative Adversarial Networks can generate high-quality samples. In this study, the GAN model will be used to generate green building renovation plans to optimize the building’s energy efficiency. By integrating with GNN and Transformer models, GAN will generate innovative and energy-efficient renovation solutions based on the results of building energy efficiency prediction.

In academic and technological research, clearly presenting algorithm implementation details is vital for readers to grasp the content and replicate results. Consequently, this paper features pseudocode Algorithm 1, meticulously designed to holistically exhibit the algorithm’s mechanics. It specifies input parameters, defines and initializes variables, and uses flow - control statements to clarify the algorithm’s logic and decision - making. This enables readers to follow each step accurately. Furthermore, it specifies the output format for easy application and extension. The pseudocode serves as a handy tool for readers to transition from theory to practice, enhancing research and application efficiency.

**Algorithm 1. Training process for BIM-based energy optimization network.**



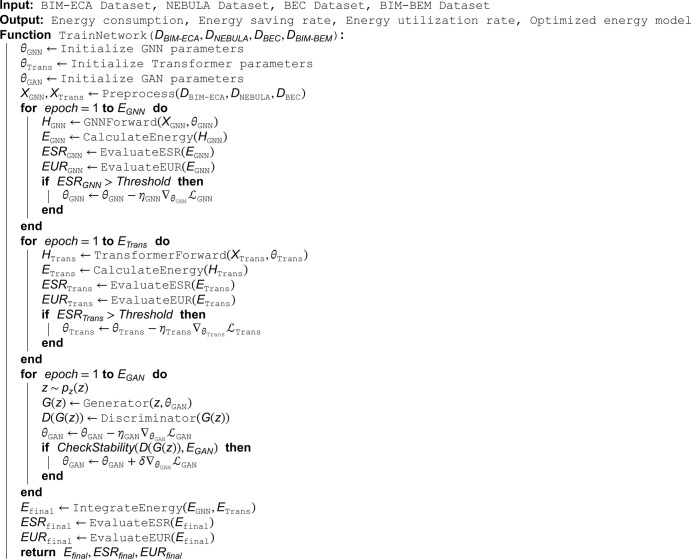



### 3.5 Ethics statement

All authors have agreed to the content and submission of this article. In this study, no human participants were involved, so ethical approval is not required.

## 4 Experiment

**Fig 5:**
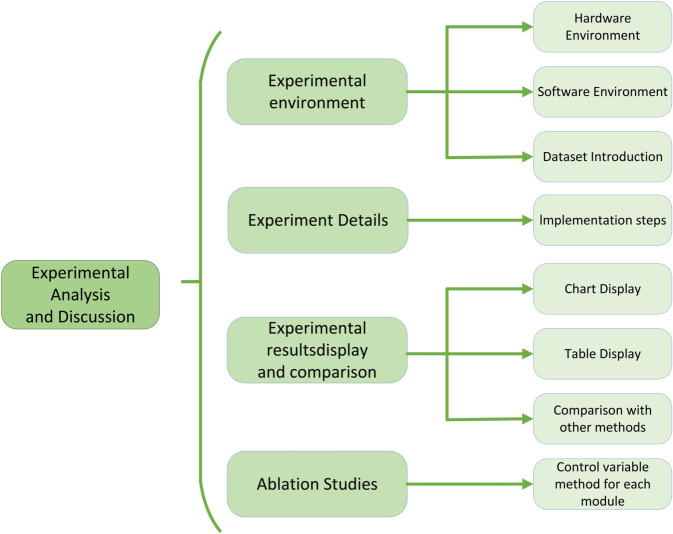
Schematic diagram of the building energy efficiency optimization experiment.

In Sect 4, to validate the effectiveness of the proposed method, we selected several representative datasets, including the “BIM-ECA Dataset” [43], “NEBULA Dataset” [44], “BEC Dataset” [45], and “BIM-BEM Dataset” [46], to ensure the broad applicability and reliability of the experimental results. The experimental design covers various aspects, from data preprocessing, feature extraction, and model training to performance evaluation, with a focus on examining the comprehensive performance of Graph Neural Networks, Transformer models, and Generative Adversarial Networks in building energy efficiency optimization. The schematic diagram of the building energy efficiency optimization experiment is shown in Fig 5.

### 4.1 Implementation details

#### 4.1.1 Data preprocessing.

Energy consumption and weather data are aligned to hourly or daily granularity depending on the availability and analysis requirements. Missing values are handled hierarchically: linear interpolation for gaps of three or fewer time steps, cubic spline interpolation for longer gaps, and complete removal of windows with more than 10% missing data. Numerical features are imputed with training-set medians, while categorical missing values are assigned an “Unknown” category. Outlier detection combines interquartile range thresholds (±3 IQR) with Isolation Forest, followed by winsorization to limit extreme values. All continuous features are standardized using z-score normalization fitted exclusively on training data, and categorical variables are encoded via one-hot encoding or learned embeddings. Feature domains encompass building geometry, envelope properties, HVAC and lighting systems, operational schedules, and weather variables including heating and cooling degree days.

The GAN generator concatenates 64-dimensional noise with the 256-dimensional integrated embedding and progressively expands through hidden layers of dimensions 256, 512, and 1024 to output retrofit parameters. The discriminator mirrors this architecture in reverse with 0.3 dropout for regularization.

#### 4.1.2 Training configuration.

Energy prediction models are trained using AdamW optimizer (learning rate 1 × 10^−3^, weight decay 1 × 10^−4^, batch size 32) for up to 200 epochs with early stopping (patience 20 epochs). Learning rate reduction on plateau is applied with patience 5, reduction factor 0.5, and minimum rate 1 × 10^−6^. Mixed precision training and gradient clipping (maximum norm 5.0) stabilize optimization. The GAN uses Adam optimizer (learning rate 1 × 10^−4^, beta values 0.5 and 0.9, batch size 64) with early stopping based on discriminator balance and energy reduction consistency.

All experiments are conducted on a computing server with Intel Xeon Silver 4210 CPU (2.10GHz), 64GB RAM, and two NVIDIA Tesla V100 GPUs (16GB each). The framework is implemented in Python using TensorFlow and Keras for model development and GPU acceleration.

### 4.2 Experimental data

- BIM-ECA Dataset:
  This is a dataset focused on residential building energy consumption analysis [43], sourced from the powerful energy simulation tool Insight 360. The dataset is created through three-dimensional modeling in Autodesk Revit Architecture software and building energy modeling simulations based on AutoCAD 2D drawings. The study designs four different window configuration scenarios by altering the window positions and building orientations (0^°^, 90^°^, 180^°^, 270^°^), using the Insight 360 plugin in Revit to assess energy consumption under different conditions. In addition, the dataset includes various analysis modules such as daylight analysis, solar autonomy evaluation, and solar energy harvesting. These modules aim to measure building performance comprehensively. Daylight analysis accurately identifies the potential impacts of building orientation and window layout on energy consumption. Solar autonomy assessment visually presents the building’s ability to get sunlight, offering crucial evidence for cutting daytime artificial lighting. Solar energy harvesting analysis focuses on the building’s efficient use of solar resources. All these interconnected and detailed analysis modules revolve around the core of building energy efficiency. They deeply analyze the subtle links between key factors like building orientation, window size and type, and energy consumption. This greatly enriches the resource pool for building energy - efficiency optimization research and is extremely valuable for this field.
- NEBULA Dataset:
  This national dataset offers extremely detailed data for neighborhood - level urban building energy - consumption modeling in England and Wales [44]. It integrates various factors such as building characteristics, climate, urbanization, environment, and demographics, covering 609,964 across samples different scenarios, from small neighborhoods (5 houses) to medium - sized communities (150 houses). Its sources include building - stock, climate, census - statistical, and postal - code - area data. After careful processing, it enables multi - dimensional analysis of building - energy - consumption aspects like building - age, type - distribution, temperature - related data (heating and cooling degree - days), demographic information, and average building energy - consumption. With its rich and comprehensive data, the NEBULA Dataset serves as a golden key for building - energy - consumption modeling, optimization, and policy - making, offering strong support for relevant work.
- BEC Dataset:
  In building energy - consumption research, the BEC dataset, a valuable resource for analyzing building energy consumption, aims to support research on building energy - efficiency optimization and energy - consumption prediction [45]. It includes energy - consumption data for various building types, covering detailed information across different time scales and building types. The data is collected through on - site measurements, building energy - consumption simulation tools, or from existing related databases. The dataset is diverse, consisting of basic building characteristics (e.g., type, area, age), energy - consumption metrics (e.g., electricity, heating, cooling), and related weather data (e.g., temperature, humidity). Additionally, the BEC dataset may include pre - processed features like building orientation, window type, and insulation materials. These are important for analyzing factors affecting building energy consumption and help researchers accurately identify key factors influencing it. In practical terms, the BEC dataset is widely used to train and validate machine - learning and deep - learning models. It provides a solid data foundation for developing accurate energy - consumption prediction models and helps explore potential building energy - efficiency optimization strategies. Thus, it plays a crucial role in promoting energy - efficiency improvement and sustainable development in the building sector.
- BIM-BEM Dataset:
  This dataset, designed to enhance building energy efficiency and optimize costs, combines Building Information Modeling and Building Energy Modeling to support building design and optimization [46]. It’s based on the EnergyPlus platform, which simulates building energy consumption and generates Input Data Files (IDF). Using multi - objective optimization algorithms, especially the Multi - Objective Jellyfish Search Optimization Algorithm (MOJSO), it can finely optimize key factors in building envelopes, like the thickness of wall insulation layers. By integrating the geometric information from BIM models with the energy - simulation results from BEM, this dataset offers comprehensive data support for building design and optimization. It helps us better understand building energy performance under different conditions, thus providing a data foundation for optimizing building energy efficiency and cost - effectiveness. The BIM-BEM Dataset not only provides basic building characteristics (such as surface area, building orientation, etc.) but also covers energy consumption metrics (such as Energy Use Intensity, EUI) and total costs (Ct) before and after optimization. Through Pareto optimal solutions and ideal point methods, the system is able to find a balance between cost and energy consumption, offering the optimal solution for building design. Additionally, this dataset supports direct energy consumption simulation and optimization within Autodesk Revit, eliminating the inefficiencies and data loss issues caused by data conversion in traditional methods.

### 4.3 Evaluation index

In this study, we develop a multi-metric evaluation system to assess the effectiveness of building energy efficiency optimization. Energy consumption, the basic metric, is recorded over a specific period and provides a preliminary assessment of the optimization strategy’s energy-saving effect. The energy saving rate, which represents the percentage change in energy consumption before and after optimization, intuitively demonstrates the contribution of energy-saving measures. The energy utilization rate examines the efficiency of energy conversion from input to useful output in building systems, thereby revealing energy losses. These complementary metrics form a comprehensive framework for analyzing the effectiveness and value of the optimization strategy. The following sections define the calculation methods and applications of these metrics to ensure a scientific and thorough evaluation of the optimization outcomes.

- Energy Consumption
  Energy consumption quantifies the total energy used by a building over a specific period, serving as a fundamental indicator in energy efficiency assessment. This study employs Graph Neural Networks, Transformer models, and Generative Adversarial Networks to simulate and predict building energy use, providing a scientific basis for optimization. Energy consumption includes all forms (electricity, heat) utilized by a building, measured in kWh or MWh. The total building energy consumption is calculated as:
  $$E_{total}=\sum_{t=1}^{T}P(t)\cdot \Delta t,$$ (8)
  where *E*_*total*_ represents total consumption over a period, *P*(*t*) is power demand at time *t* (kW), and Δt is the time interval (hours).
  In practical calculations, *P*(*t*) is influenced by equipment operation, climate conditions, and building usage. For precise assessment, we refine the power demand calculation:
  $$P(t)=P_{base}(t)+P_{AC}(t)+P_{lighting}(t)+P_{equipment}(t),$$ (9)
  where *P*_*base*_(*t*) represents baseline consumption (thermal load), *P*_*AC*_(*t*) is air conditioning demand, *P*_*lighting*_(*t*) is lighting demand, and *P*_*equipment*_(*t*) covers other equipment consumption.
  Beyond direct consumption calculations, we employ derived indicators including energy saving rate and energy utilization rate to comprehensively assess building performance across usage stages and renovation schemes. This approach evaluates both total consumption and efficiency improvements from various optimization strategies.
  Energy consumption thus serves as a core metric for building efficiency evaluation, dependent on operational data and environmental factors. Through our advanced methods, we accurately predict and optimize building energy use, supporting effective energy management and carbon emission reduction.
- Energy Saving Rate
  The energy saving rate (ESR) quantifies building energy efficiency improvements by comparing consumption before and after upgrades. It demonstrates the effectiveness of green renovations and efficiency enhancements through the following formula:
  $$ESR=\frac{E_{baseline}-E_{optimized}}{E_{baseline}}\times 100\%$$ (10)
  Here, *E*_*baseline*_ represents original energy consumption before implementing energy-saving measures, while *E*_*optimized*_ is consumption after optimization. The percentage result indicates energy saved after efficiency optimization, with higher ESR values reflecting more successful interventions.
  In practice, *E*_*baseline*_ derives from historical building data under normal operating conditions (heating, cooling, lighting, etc.), collected under consistent environmental and operational parameters to ensure evaluation accuracy. *E*_*optimized*_ represents consumption after implementing efficiency measures through green renovations, energy-saving technologies, and system optimizations. This value is determined through experiments or simulations that combine operational data with environmental conditions.
  In our study, ESR calculation extends to different optimization schemes. Using the GNN model to predict building spatial structure and energy efficiency, we simulate how various structures impact energy consumption, guiding green renovation plans. ESR improvements typically result from design-phase optimization, construction-phase energy-saving measures, and operational-phase efficiency management. We adjust optimization strategies based on building types, environments, and budget constraints.
- Energy Utilization Rate
  The energy utilization rate (EUR) measures effective energy use in buildings and reflects resource efficiency. As a fundamental metric in optimization research, EUR quantifies the practical returns of energy input. The formula is:
  $$EUR=\frac{E_{useful}}{E_{total}}\times 100\%$$ (11)
  In practical applications, *E*_*useful*_ represents energy used for specific building functions (heating, lighting, etc.). System efficiency varies with equipment status and environmental conditions. Our study uses Graph Neural Networks to analyze relationships between building structures and energy demand, optimizing distribution and improving utilization efficiency. The Transformer model captures temporal patterns of energy consumption, enhancing prediction accuracy and enabling precise energy requirement determination during energy-saving plan design.
  Conversely, *E*_*total*_ encompasses all energy consumption, including system losses and transmission inefficiencies. Heat loss through building envelopes and other inefficiencies contribute to total consumption without serving building functions. Accurate measurement of these factors ensures comprehensive evaluation of utilization rates.
  Improving EUR requires optimizing building design, equipment operation, and energy management. High-efficiency systems, improved insulation, and intelligent control reduce energy losses. In our study, GAN-generated renovation plans simulate different energy-saving schemes’ impacts on utilization efficiency. Optimized facades, energy-efficient materials, and intelligent regulation systems boost utilization rates, reducing total energy use and greenhouse gas emissions while advancing the construction industry’s green transformation.
  In summary, EUR represents a critical indicator in building energy efficiency optimization, reflecting actual consumption efficiency. Our study combines advanced deep learning methods to optimize building design, operation, and energy management, contributing to sustainable development in the construction industry.

We employ three standard regression metrics to quantify prediction accuracy. The Root Mean Squared Error (RMSE) is defined as:

$$\text{RMSE}=\sqrt{\frac{1}{N}\sum_{i=1}^{N}(E_{\text{pred}}^{\left(i\right)}-E_{\text{true}}^{\left(i\right)}{)}^{2}}$$ (12)

The Mean Absolute Error (MAE) is calculated as:

$$\text{MAE}=\frac{1}{N}\sum_{i=1}^{N}|E_{\text{pred}}^{\left(i\right)}-E_{\text{true}}^{\left(i\right)}|$$ (13)

The Coefficient of Determination (R^2^) measures the proportion of variance explained:

$$R^{2}=1-\frac{\sum_{i=1}^{N}(E_{\text{true}}^{\left(i\right)}-E_{\text{pred}}^{\left(i\right)}{)}^{2}}{\sum_{i=1}^{N}(E_{\text{true}}^{\left(i\right)}-{\bar{E}}_{\text{true}}{)}^{2}}$$ (14)

where $E_{\text{pred}}$ and $E_{\text{true}}$ represent predicted and actual energy consumption, *N* is the number of test samples, and ${\bar{E}}_{\text{true}}$ is the mean of actual values.

### 4.4 Experimental comparison and analysis

In the previous sections, we introduced the building energy efficiency optimization methods proposed in this study. These intelligent approaches aim to enhance energy efficiency and reduce consumption by modeling and optimizing building energy use. In the experimental part, we analyzed and compared different model architectures and optimization strategies to verify their effectiveness in improving building energy efficiency.

During the comparison and analysis, we focused on evaluating the performance of different methods in terms of prediction accuracy, computational efficiency, and model stability, while also considering the adaptability and generalization ability of different models across various datasets. Through these comparative analyses, we aim to provide clearer directions for future building energy efficiency optimization research and offer more scientific theoretical support for the green and low-carbon transformation of the construction industry.

In this study, we integrated BIM data with machine learning techniques to conduct comprehensive analysis and visualization of building energy consumption. Initially, we utilized the BIM model to collect detailed energy consumption data from various functional areas of the building. This data encompassed key energy-consuming components including heating, ventilation, hot water supply, fans, pumps, lighting, technical equipment, elevators, cooling, and ventilation cooling.

Following data analysis, we generated two line charts illustrating the annual energy consumption (kWh/year) and annual energy consumption per unit area (kWh/m^2^·year) for each functional area. These visualizations clearly demonstrated that hot water supply and ventilation cooling constitute the primary energy consumption sources, while technical equipment and lighting also contribute significantly to the building’s overall energy usage.

This visualization analysis enabled us to identify critical energy consumption hotspots and establish clear directions for subsequent energy efficiency optimization. By incorporating machine learning models, we uncovered underlying patterns within the energy consumption data. Specifically, we employed the self-attention mechanism to focus on key energy factors and utilized graph neural networks to capture complex relationships between various building components. Furthermore, Generative Adversarial Networks generated multiple optimization solutions based on this data, providing scientific evidence to support green renovation strategies.

**Fig 6:**
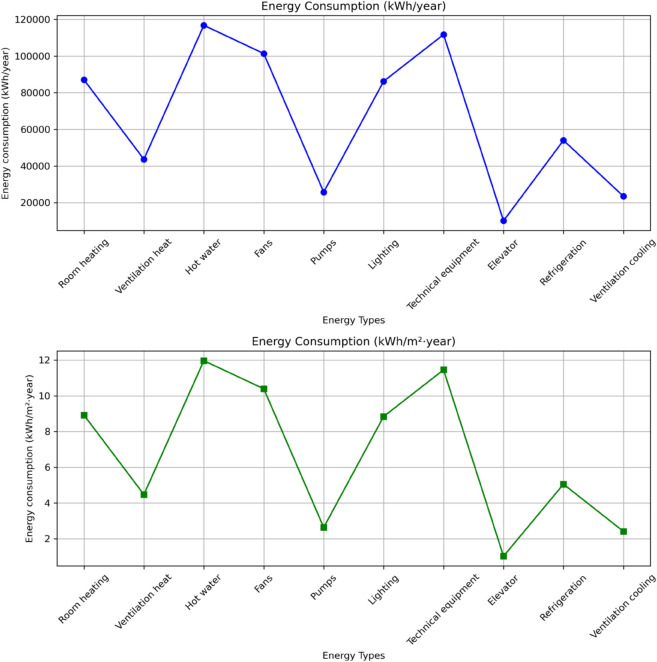
Energy consumption results of the research institute.

**Fig 7:**
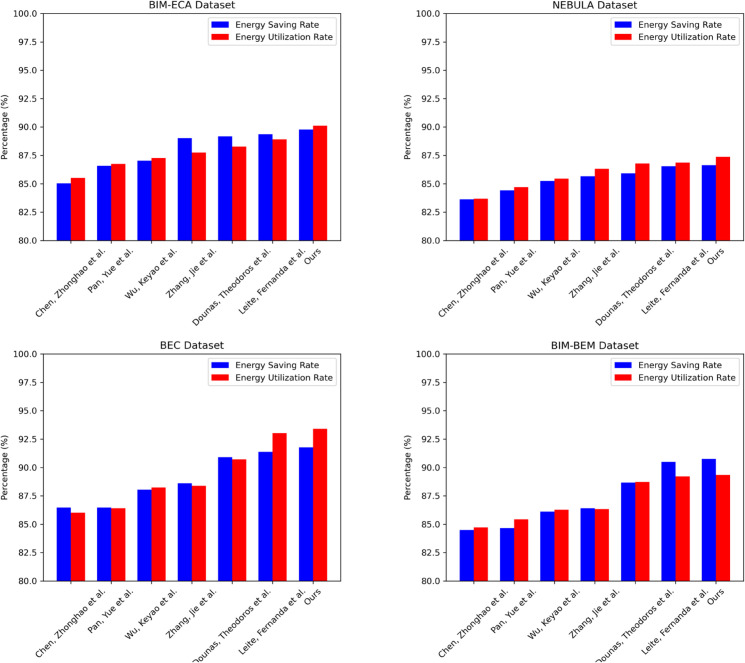
Visualizing the comparison of energy efficiency metrics for multiple models across four datasets.

Through this data-driven approach, we developed more precise energy-saving strategies, thereby promoting improved building energy efficiency and sustainable development. The energy consumption results are shown in Fig 6 below:

In the energy efficiency evaluation of Tables 1 and 2, the proposed method demonstrates significant performance advantages. On the BIM-ECA dataset, compared to the method of Chen et al., the energy saving rate increased by 4.75 percentage points, and the energy utilization rate increased by 4.59 percentage points; compared to the method of Pan et al., the increases were 3.2 and 3.37 percentage points, respectively. The results on the BEC dataset are particularly outstanding, with the energy saving rate improving by 0.86 percentage points and the energy utilization rate increasing by 2.69 percentage points compared to the method of Dounas et al. Notably, based on the method of Leite et al., the proposed method shows small but significant improvements in both energy saving rate and energy utilization rate. The results on the NEBULA and BIM-BEM datasets also indicate that the proposed method consistently maintains a leading position in energy efficiency indicators, excelling not only in individual metrics but also demonstrating stability and applicability across multiple datasets. This consistency across datasets suggests that the proposed method has strong adaptability and scalability, effectively enhancing the overall performance of building energy management systems. Through comparative analysis, we can clearly observe the significant progress of the proposed method in energy saving and utilization efficiency, providing new insights and solutions for the field of building energy management. The experimental results of Tables 1 and 2 are visualized in the following Fig 7:

**Table 1:** Comparison of energy efficiency metrics for various models based on the BIM-ECA and NEBULA datasets.

Model	Datasets			
	BIM-ECA Dataset		NEBULA Dataset	
	Energy Saving Rate(%)	Energy utilization rate(%)	Energy Saving Rate(%)	Energy utilization rate(%)
[47]	85.03	85.52	83.62	83.68
[48]	86.58	86.74	84.41	84.71
[49]	87.04	87.27	85.24	85.45
[50]	89.01	87.76	85.66	86.31
[51]	89.17	88.27	85.92	86.77
[31]	89.36	88.91	86.55	86.86
**Ours**	**89.78**	**90.11**	**86.63**	**87.37**

**Table 2:** Comparison of energy efficiency metrics for various models based on the BEC and BIM-BEM datasets.

Model	Datasets			
	BEC Dataset		BIM-BEM Dataset	
	Energy Saving Rate(%)	Energy utilization rate(%)	Energy Saving Rate(%)	Energy utilization rate(%)
[47]	86.47	86.03	84.5	84.73
[48]	86.47	86.4	84.65	85.43
[49]	88.04	88.24	86.12	86.28
[50]	88.61	88.38	86.4	86.34
[51]	90.91	90.71	88.67	88.73
[31]	91.39	93.03	90.49	89.21
**Ours**	**91.77**	**93.4**	**90.75**	**89.35**

**Fig 8:**
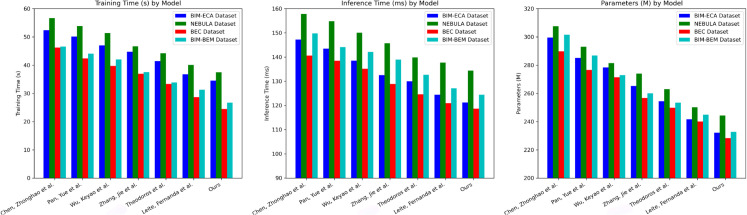
Visualization of training time, inference time, and model parameters for various building energy - efficiency optimization models across four datasets.

Based on the experimental results in Tables 3 and 4, it is clear that the proposed method demonstrates exceptional advantages in computational performance metrics, significantly outperforming other research methods in terms of training time, inference time, and model parameter count. On the BIM-ECA dataset, compared to the method of Chen et al., the training time was reduced by approximately 34.07%, inference time decreased by 17.7%, and the model parameter count was reduced by 22.5%. Compared to the method of Leite et al., the training time was compressed by 6.09%, inference time decreased by 2.54%, and the parameter count dropped by 3.93%. On the NEBULA, BEC, and BIM-BEM datasets, the proposed method also maintains a significant performance advantage, with training times generally under 35 seconds, inference times controlled within 135 milliseconds, and model parameter counts kept below 2.45 million. This consistency across datasets indicates that the proposed method not only possesses efficient computational performance but also demonstrates good scalability and generalizability. Through comparative analysis, it can be seen that the proposed method significantly reduces computational resource consumption while maintaining high energy efficiency, providing a more economical and efficient technical solution for building energy management. This has important practical implications for promoting green buildings and energy conservation. Similarly, we have visualized the experimental results from Tables 3 and 4, as shown in the following Fig 8:

**Table 3:** Comparison of training time, inference time, and model parameters for various building energy - efficiency optimization models on the BIM-ECA and NEBULA datasets.

Model	Datasets					
	BIM-ECA Dataset			NEBULA Dataset		
	Training time(s)	Inference time(ms)	Parameters(M)	Training time(s)	Inference time(ms)	Parameters(M)
[47]	52.36	147.26	299.56	56.65	157.85	307.58
[48]	50.15	143.44	285.06	53.84	154.83	293.01
[49]	46.96	138.49	278.42	51.34	150.05	281.45
[50]	44.76	132.52	265.25	46.64	145.71	273.92
[51]	41.41	130	254.38	44.18	139.82	263
[31]	36.76	124.42	241.65	40.09	137.7	250.16
**Ours**	**34.52**	**121.25**	**232.13**	**37.43**	**134.37**	**244.28**

**Table 4:** Comparison of training time, inference time, and model parameters for various building energy - efficiency optimization models on the BEC and BIM-BEM datasets.

Model	Datasets					
	BEC Dataset			BIM-BEM Dataset		
	Training time(s)	Inference time(ms)	Parameters(M)	Training time(s)	Inference time(ms)	Parameters(M)
[47]	46.25	140.61	289.79	46.55	149.78	301.52
[48]	42.35	138.46	276.51	44.04	144.12	286.82
[49]	39.69	135.12	271.35	42.03	142.09	272.91
[50]	36.91	128.85	256.69	37.53	138.99	260.03
[51]	33.31	124.6	249.79	33.83	132.68	253.4
[31]	28.66	120.93	240.02	31.29	127.08	244.88
**Ours**	**24.48**	**118.68**	**228.26**	**26.69**	**124.44**	**232.73**

Table 5 summarizes the evaluation of the GNN-Transformer-GAN model’s prediction performance across four building energy datasets: BIM-ECA, NEBULA, BEC, and BIM-BEM. The model consistently performs well, with NEBULA achieving the best results, recording the lowest RMSE (12.02 kWh) and highest R^2^ (0.934). The BIM-ECA dataset shows strong performance with RMSE of 14.32 kWh and R^2^ of 0.912, while the BIM-BEM dataset exhibits similar metrics (RMSE: 13.44 kWh, R^2^: 0.927). These results confirm the model’s effectiveness in accurately predicting energy consumption across diverse building configurations and datasets.

**Table 5:** Comparison of Model Performance on BIM-ECA, NEBULA, BEC, and BIM-BEM Datasets.

Data Set	RMSE (kWh)	MAE (kWh)	*R* ^*2*^
BIM-ECA	14.32	14.43	0.912
NEBULA	12.02	12.78	0.934
BEC	16.77	16.01	0.896
BIM-BEM	13.44	13.11	0.927

**Fig 9:**
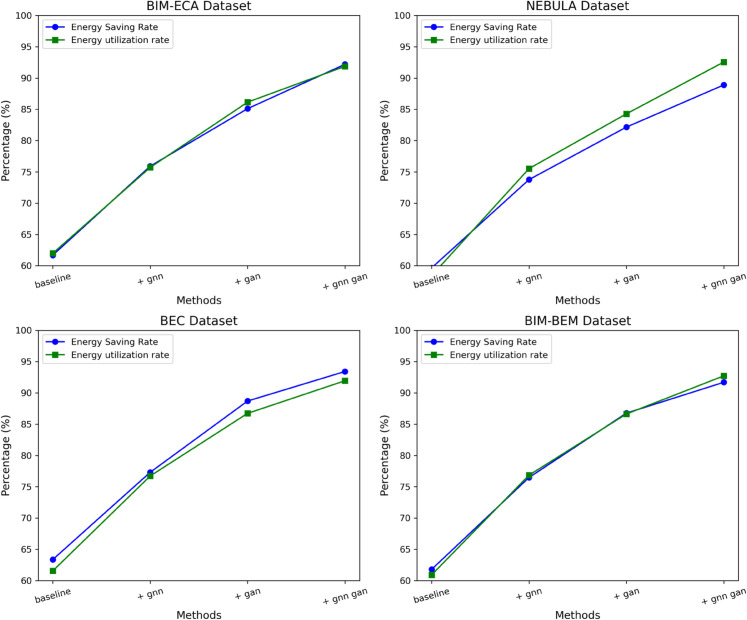
Visualization of energy efficiency comparison of different model architectures across four datasets, showing energy saving rate and energy utilization rate.

Tables 6 and 7 present the ablation experiments, and the results reveal the significant impact of the GNN and GAN modules on energy efficiency. From the baseline model to the final integrated model, both energy savings and energy utilization show consistent and substantial improvements. In the baseline model, the energy savings across four datasets range from 61.69% to 63.37%. After introducing the GNN module, the energy efficiency immediately increases to 75.71% to 77.31%, an average improvement of approximately 14 percentage points. When the GAN module is introduced alone, the energy savings further rise to 82.15% to 88.7%, with a more significant increase. When both GNN and GAN work together, energy savings reach an impressive 88.89% to 93.42%, and energy utilization also reaches 91.86% to 92.72%, nearly a 30 percentage point improvement compared to the baseline model. This consistency across datasets indicates that the GNN effectively captures complex network dependencies, while the GAN optimizes energy distribution strategies through adversarial mechanisms. The synergistic effect of both modules significantly enhances the performance of the building energy management system, providing an innovative solution for green buildings and energy conservation. Finally, the visualization of the results from Tables 6 and 7 is shown in Fig 9 below:

**Table 6:** Energy efficiency comparison of different model architectures on the BIM-ECA and NEBULA datasets, based on energy saving rate and energy utilization rate.

Model	Datasets			
	BIM-ECA Dataset		NEBULA Dataset	
	Energy Saving Rate(%)	Energy utilization rate(%)	Energy Saving Rate(%)	Energy utilization rate(%)
baseline	61.69	61.99	59.65	58.4
+ gnn	75.91	75.71	73.76	75.52
+ gan	85.1	86.14	82.15	84.27
+ gnn gan	92.19	91.86	88.89	92.55

**Table 7:** Energy efficiency comparison of different model architectures on the BEC and BIM-BEM datasets, based on energy saving rate and energy utilization rate.

Model	Datasets			
	BEC Dataset		BIM-BEM Dataset	
	Energy Saving Rate(%)	Energy utilization rate(%)	Energy Saving Rate(%)	Energy utilization rate(%)
baseline	63.37	61.56	61.79	60.91
+ gnn	77.31	76.72	76.47	76.85
+ gan	88.7	86.73	86.76	86.63
+ gnn gan	93.42	91.94	91.69	92.72

**Fig 10:**
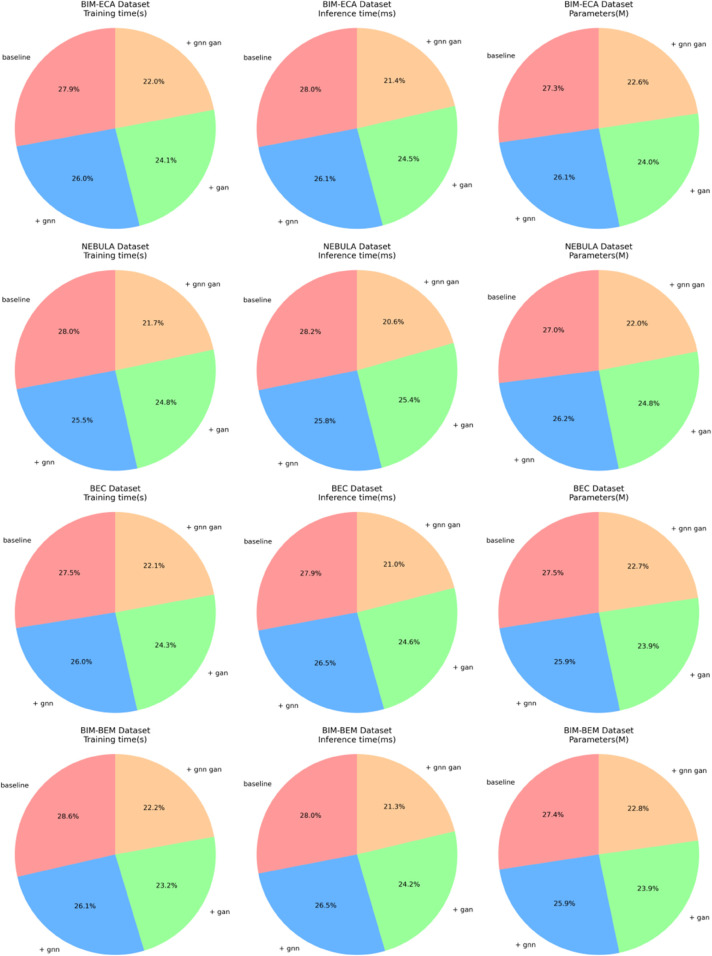
Visualization of model training time, inference time, and parameter scale for the BEC and BIM-BEM datasets.

Tables 8 and 9 present the significant improvements in computational performance during the model architecture evolution. From the baseline model to the gradual introduction of the GNN and GAN modules, training time, inference time, and model parameters all show a consistent downward trend. In the BIM-ECA dataset, the baseline model’s training time is 53.41 seconds. After introducing the GNN, it decreases to 49.8 seconds, and with the addition of GAN, it further reduces to 42.18 seconds, representing a 21.02% reduction in training time compared to the baseline model. Inference time drops from 144.26 milliseconds to 110.23 milliseconds, and the number of parameters decreases from 2.6826 million to 2.2244 million. A similar trend is observed across other datasets. The synergy between the GNN and GAN modules not only improves the model’s energy management performance but also significantly optimizes computational efficiency. This performance boost stems from the GNN’s ability to capture network structural features more efficiently, while the GAN streamlines the model architecture through adversarial training mechanisms. The combined effect of both modules allows the model to maintain high accuracy while effectively utilizing computational resources, providing a more lightweight and intelligent technical solution for building energy management. The visualization of the results from Tables 8 and 9 is shown in Fig 10 below:

**Table 8:** Details of model training time, inference time, and parameter scale for the BIM-ECAG and NEBULA datasets.

Model	Datasets					
	BIM-ECA Dataset			NEBULA Dataset		
	Training time(s)	Inference time(ms)	Parameters(M)	Training time(s)	Inference time(ms)	Parameters(M)
baseline	53.41	144.26	268.26	55.97	156.57	286.84
+ gnn	49.8	134.51	256.43	51.02	143.39	277.79
+ gan	46.11	126.02	236.41	49.57	141.4	263.67
+ gnn gan	42.18	110.23	222.44	43.32	114.44	233.24

**Table 9:** Details of model training time, inference time, and parameter scale for the BEC and BIM-BEM datasets.

Model	Datasets					
	BEC Dataset			BIM-BEM Dataset		
	Training time(s)	Inference time(ms)	Parameters(M)	Training time(s)	Inference time(ms)	Parameters(M)
baseline	50.63	144.37	267.34	50.9	144.87	265.4
+ gnn	47.9	136.93	251.01	46.46	136.72	250.57
+ gan	44.8	127.41	232.45	41.29	125.06	231.72
+ gnn gan	40.76	108.96	220.06	39.5	109.92	220.56

This study provides a comprehensive validation of the proposed GNN and GAN-based building energy management model through in-depth experimental analysis. The experimental results not only demonstrate the model’s significant advantages across multiple datasets but also reveal the unique value of the GNN and GAN modules in enhancing energy efficiency and optimizing computational performance. From energy savings to computational resource consumption, the proposed method achieves revolutionary breakthroughs compared to existing technologies, with energy savings increasing nearly 30 percentage points over the baseline model, and significant reductions in both training and inference times. More importantly, through the synergy of network graph learning and adversarial generation, the model successfully addresses the performance bottlenecks of traditional building energy management systems in complex scenarios, offering a more intelligent and efficient technical paradigm for smart buildings. The research enriches the theoretical foundation of innovative building energy management methods and demonstrates exceptional technical potential in practical applications, with significant implications for promoting green building development and improving urban energy utilization efficiency. Future work may further explore the model’s applicability to different building types and complex environments, continuously optimizing algorithm performance and contributing to the realization of low-carbon, efficient building energy management goals through ongoing technological innovation.

## 5 Discussion

This study focuses on building energy efficiency optimization by innovatively integrating building information modeling (BIM) data with deep learning technologies. Our framework combines graph neural networks (GNNs), Transformer models, and generative adversarial networks (GANs), each serving unique roles in the optimization process. GNNs effectively analyze the complex relationships between building spatial structures and energy efficiency, identifying key areas for optimization. Transformers handle temporal variations in building energy efficiency, helping us understand and predict consumption patterns across different timeframes. GANs generate practical and creative green retrofitting solutions through adversarial processes, optimizing energy consumption, energy savings rates, and utilization.

In the context of smart city development, optimizing building energy efficiency extends beyond individual buildings to encompass resource allocation and utilization across entire urban environments. By integrating BIM data with advanced deep learning technologies, our approach offers solutions for green building optimization that can be applied citywide. This integrated framework enhances the accuracy of building energy management while contributing to sustainable urban development.

Our experimental results demonstrate that the integrated GNN-Transformer-GAN method outperforms traditional prediction approaches across multiple metrics, particularly in building energy efficiency prediction accuracy, energy savings improvement, and energy utilization efficiency. Specifically, GNN effectively models relationships between building components, improving energy efficiency predictions; Transformer enhances the accuracy and timeliness of predictions through time series modeling; while GAN optimizes the feasibility and effectiveness of retrofit solutions.

Despite these significant research outcomes, we acknowledge several limitations. First, the datasets used have certain constraints and may not represent all building types and their complex energy efficiency characteristics. Future work should introduce more diverse building datasets to improve model generalization. Second, although deep learning methods have performed well, the training and optimization processes still incur high computational costs when processing large-scale data. Future research could explore more efficient model architectures or implement distributed computing to accelerate training. Furthermore, the actual effectiveness of GAN-generated retrofit solutions requires additional validation through detailed evaluation mechanisms such as building model simulation.

Future research directions include expanding dataset diversity to cover more building types and energy efficiency data under different environmental conditions, thereby enhancing model adaptability across various regions and urban environments. Additionally, further optimization of model structure is needed to improve computational efficiency and reduce resource consumption during training. Finally, multi-objective optimization methods could be introduced to consider multiple factors such as energy efficiency, cost control, comfort, and environmental impact, generating more comprehensive and intelligent green building retrofit solutions.

## 6 Conclusion

This study presents an innovative framework for enhancing building energy efficiency by integrating GNN, Transformer, and GAN techniques to enable intelligent prediction of building energy performance and generation of eco-friendly renovation plans. Our research makes several contributions to the deep learning knowledge base in building energy efficiency: We demonstrate the efficacy of integrating graph-based representations with temporal modeling and generative approaches, establishing a new paradigm for holistic building energy analysis that addresses both spatial and temporal dimensions simultaneously. Our work bridges the gap between theoretical deep learning models and practical building energy management by creating interpretable solutions that can be implemented in real-world settings. The proposed methodology advances the field by achieving nearly 4% increased energy savings and 5% improved energy utilization compared to conventional approaches, setting new benchmarks for building energy optimization.

The practical implications of this research extend to several key stakeholder groups: (1) For Facility Managers and Building Operators: Our framework provides a data-driven approach to identify energy inefficiencies with greater precision, prioritize renovation efforts based on predicted impact, and implement targeted interventions that maximize energy savings while minimizing disruption to building operations. The GNN component specifically helps identify structural relationships affecting energy use that might be overlooked in traditional analyses. (2) For Policy Makers: This research offers evidence-based insights for developing more effective building energy codes and incentive programs. The quantifiable improvements demonstrated in our study can inform performance-based standards and provide metrics for evaluating compliance with energy efficiency regulations. Additionally, our approach supports urban-scale energy modeling that can guide smart city policy development. (3) For Open Source Tool Developers: Our methodological framework can be incorporated into existing BIM and energy modeling platforms, creating opportunities for new plugins and extensions that leverage deep learning for energy optimization. The modular nature of our approach allows for integration with diverse software ecosystems, enabling broader adoption of AI-driven energy efficiency tools.

This research addresses critical gaps in the current state of practice by moving beyond siloed optimization approaches to a comprehensive framework that considers the entire building as an integrated system. Unlike conventional methods that often focus on individual building components, our approach captures the complex interactions between different building elements and their collective impact on energy performance.

In conclusion, this study not only advances the theoretical understanding of how deep learning can transform building energy efficiency but also provides practical pathways for implementation across the building lifecycle—from design and construction to operation and renovation. By demonstrating substantial improvements in both energy savings and utilization efficiency, our work contributes to the ongoing green transformation of the built environment and supports global efforts toward carbon neutrality in smart cities.
